# A start-up of psychrophilic anaerobic sequence batch reactor digesting a 35 % total solids feed of dairy manure and wheat straw

**DOI:** 10.1186/s13568-015-0144-1

**Published:** 2015-08-20

**Authors:** Noori M. Cata Saady, Daniel I. Massé

**Affiliations:** Dairy and Swine Research and Development Centre, Agriculture and Agri-Food Canada, Sherbrooke, QC J1M 0C8 Canada

**Keywords:** Dry anaerobic digestion, Cow manure, Methane, Psychrophilic, Wheat straw, Start-up

## Abstract

Zero liquid discharge is currently an objective in livestock manure management to minimize water pollution. This paper reports the start-up phase of a novel psychrophilic (20 °C) dry anaerobic digestion of dairy manure with bedding fed at 35 % total solids and an organic loading rate of 3.0 g total chemical oxygen demand kg^−1^ inoculum day^−1^ in anaerobic sequence batch reactors. The specific methane (CH_4_) yield ranged from 165.4 ± 9.8 to 213.9 ± 13.6 NL CH_4_ kg^−1^ volatile solids (VS) with an overall average of 188 ± 17 NL CH_4_ kg^−1^ VS during 11 successive start-up cycles (231 days) and a maximum CH_4_ production rate of 10.2 ± 0.6 NL CH_4_ kg^−1^ VS day^−1^. The inoculum-to-substrate (VS-based) ratio ranged from 4.06 to 4.47. Although methanogenesis proceeded fairly well the hydrolysis seemed to be the rate limiting step. It is possible start up psychrophilic dry anaerobic digestion of cow feces and wheat straw at feed TS of 35 % within 7–10 successive cycles (147–210 days).

## Introduction

Environmental regulations aim to decrease the adverse impact of agricultural activities on the natural resources and environment. In this context, livestock industry produces large amount of manure that needs to be treated, stabilized, and converted to fertilizers; one opportunity to extract energy from livestock manure is the use of anaerobic digestion. In Canada and USA, cattle generate about 75 and 86 % of the manure produced by livestock, respectively (Hofmann and Beaulieu 2006; Wen et al. 2004). Although fresh cow feces has a total solids (TS) content of about 12–14 %, the addition of bedding materials such as straw increases the total solids to more than 25 % (El-Mashad et al. 2004). Conventional wet anaerobic digestion (WAD) is currently used to stabilize livestock manure, extract renewable energy, reduce its environmental impact, and convert it to fertilizers. However, WAD requires large reactors volume because influent has low total solids content (<10 %) (El-Mashad et al. 2004).

Recently, dry anaerobic digestion (DAD) has gained more interest. The advantages of DAD have been demonstrated at mesophilic and thermophilic conditions for the organic fraction of municipal solid wastes (15 % TS) (Challen Urbanic et al. 2011; Li et al. 2011b; Ramasamy and Abbasi 2000) and for agricultural wastes and livestock manure (15–20 % TS) (Ahn et al. 2010; Kusch et al. 2008; Di Maria et al. 2012). However, in cold-climate regions psychrophilic operation is desired. Obviously, heating the bioreactor to maintain mesophilic and thermophilic conditions is an energy drain. Kashyap et al. (2003) indicated that developing a psychrophilic anaerobic digestion process to convert cattle dung into biogas and meet the energy needs at cold-climate regions is still a technological challenge; since then on-farm psychrophilic WAD has been developed and deployed (Massé et al. 1996, 1997, 2007, 2010). Presently, developing and optimizing a psychrophilic dry anaerobic digestion process is of outmost importance and could fill a gap in the anaerobic digestion market.

Psychrophilic conditions decrease the rates of chemical and biological reactions compared to mesophilic and thermophilic operations. According to microbial thermodynamic, a drop in temperature increases the energy required to enable the endothermic reactions of propionate and butyrate conversion to acetate. Similarly, temperature drop decreases the energy released from spontaneous exothermic reactions except for hydrogenotrophic microorganisms (Lettinga et al. 2001). The low rate of specific growth at psychrophilic condition restricts the lower threshold limit of the microorganisms’ retention time to prevent biomass washout in continuous systems and to achieve reasonable degree of treatment in batch systems. Generally, the start-up of psychrophilic anaerobic digestion requires long time to enable retention of sufficient slow growing microbial biomass (Dhaked et al. 2010).

Recently, a psychrophilic (20 °C) dry anaerobic digestion (PDAD) of cow feces and wheat straw in sequential batch reactor (SBR), has been developed at Agriculture and Agri-Food, Dairy and swine Research and Development Centre (DSRDC) in Sherbrooke, QC, Canada to stabilize cow manure and convert it to biofuel (Massé and Saady 2015a, b, c; Saady and Massé 2015).

Massé and Saady (2015b) reported long term operation (252 days) of PDAD-SBR for digesting cow feces (TS 13–16 %) with an average specific methane yield (SMY) of 222 ± 27.2 NL CH_4_ kg^−1^ VS fed. Moreover, they demonstrated successful operation at TS of 27 % and organic loading rate (OLR) of 3 g total chemical oxygen demand (TCOD) kg^−1^ inoculum day^−1^ for 273 days with an average SMY of 182.9 ± 16.9 NL CH_4_ kg^−1^ VS fed (Massé and Saady 2015c). They feasibility of PDAD-SBR of cow feces and wheat straw (27 % TS in feed) has been demonstrated at OLR of 4.0, 5.0, and 6.0 g TCOD kg^−1^ inoculum day^−1^ in long term study (315 days) with SMYs of 187.3 ± 18.1, 163.6 ± 39.5, 150.8 ± 32.9 NL CH_4_ kg^−1^ VS fed, respectively (Saady and Massé 2015). The OLR of cow feces and wheat straw (27 % TS in feed) fed to PDAD-SBR has been increased to 7.0 and 8.0 g TCOD kg^−1^ inoculum day^−1^ in long term study (84 days) and SMYs of 147.1 ± 17.2, 143.2 ± 11.7 NL CH_4_ kg^−1^ VS fed, respectively, have been reported (Massé and Saady 2015a).

Increasing the total solids content of the substrate fed to a DAD bioreactor is a basic engineering design objective to decrease the bioreactor volume (Luning et al. 2003), reduce its construction costs, and increase the specific energy output per reactor’s unit volume. However, Motte et al. (2013) concluded that mesophilic acidogenesis of wheat straw decreased with the increase in its TS from 10 to 33 % but with no changes in the metabolic pathway until a clear limit at 28 % TS.

The principal objective of this study was to assess the feasibility of starting-up a psychrophilic (20 °C) dry anaerobic digestion of the dairy cow feces and wheat straw at 35 % TS in feed in terms of specific methane yield, volatile solids removal, process stability and start-up duration.

## Materials and methods

### Experimental setup

The experiments assessed the start-up strategy and duration of cow feces and wheat straw psychrophilic anaerobic digestion at feed total solids of 35 % and an OLR of 3.0 g TCOD kg^−1^ inoculum day^−1^. The operation comprised of 231 days involving eleven successive cycles. The treatment cycle length (TCL) was 21 days. The cycle includes the following steps: day 1: loading the reactor with inoculum, feeding the substrate, and mixing the inoculum and substrate; days 1–21: reaction; and day 21: unloading the digestate and starting the next cycle.

At the beginning of each cycle, the contents of the triplicate reactors were mixed and homogenized, then 6 kg of the mixture was used as an inoculum in each of the reactors, then each reactor received the amount of cow feces and wheat straw according to the OLR and TS desired, then the content of the bioreactor was mixed manually for 5 min and a representative sample (50 g) has been taken. Thereafter the reactor has been flushed with nitrogen to maintain anaerobic digestion. The mass of inoculum, feces, and/or straw fed to each bioreactor at the beginning of the successive cycles, the VS-based inoculum-to-substrate ratio (ISR) and the OLR are given in Table 1. All the feeding calculations have been based on target OLR as well as the masses of inoculum and the composition of cow feces and wheat straw. The digestion cycle was conducted in a static mode with no leachate recirculation. Physico-chemical characteristics of the inoculum and substrates (manure and straw) before feeding bioreactors were analyzed and are given in Table 2.

**Table 1:** Organic loading rate and total solids of the feed

Cycle	Feces (kg)	Straw (kg)	ISR (VS-based ratio)	Organic loading Organic loading rate			
				TCOD fed (g)	VS fed (g)	g TCOD substrate kg^−1^ inoculum day^−1^	g VS substrate kg^−1^ inoculum day^−1^
1	0.70	0.30	4.47	378	334.8	3.0	2.66
2	0.635	0.259	4.06	378	295.7	3.0	2.35
3	0.545	0.252	4.30	378	279.1	3.0	2.22
4	0.567	0.259	4.18	378	287.1	3.0	2.28
5	0.567	0.259	4.18	378	284.2	3.0	2.26
6	0.567	0.259	4.18	378	284.2	3.0	2.26
7	0.606	0.247	4.32	378	278.4	3.0	2.28
8	0.606	0.247	4.32	378	277.8	3.0	2.20
9	0.606	0.247	4.32	378	277.8	3.0	2.20
10	0.634	0.217	4.36	378	275.1	3.0	2.18
11	0.634	0.217	4.36	378	275.1	3.0	2.18

In all cycles the reactors have been inoculated with 6 kg of culture which has been transferred from the previous cycle. The feed TS has been kept at 35 % in all cycles

**Table 2:** Physicochemical characteristics of the inoculum, cow feces and inoculum-substrate mixture at beginning of digestion cycle

Cycle	Type	Acetate (g kg^−1^)	Propionate (g kg^−1^)	Butyrate (g kg^−1^)	pH	Alkalinity (g kg^−1^ as CaCO_3_)	TCOD (g kg^−1^)	TS (%)	VS (%)
1	Inoculum	0.77 ± 0.47	0.24 ± 0.21	0.28 ± 0.08	7.3 ± 0.0	11.5 ± 0.55		11.0 ± 0.6	9.3 ± 0.5
	Feces	4.5	1.1	0.93	5.89		148.1	13.29	11.9
	ISM	1.1 ± 0.02	0.25 ± 0.02	0.29 ± 0.02	7.1 ± 0.0			14.1 ± 0.4	12.0 ± 0.6
2	Inoculum	0.84 ± 0.50	0.32 ± 0.22	0.26 ± 0.07	7.4 ± 0.1	11.8 ± 0.65		12.3 ± 0.4	10.6 ± 0.4
	Feces	3.4	0.88	1.39	6.18		148.1	13.29	11.9
	ISM	1.2 ± 0.05	0.28 ± 0.02	0.10 ± .01	7.0 ± 0.1			15.3 ± 0.1	13.4 ± 0.1
3	Inoculum	0.25 ± 0.03	0.0 ± 0.0	0.0 ± 0.0	7.3 ± 0.1	10.0 ± 1.8		13.8 ± 0.7	11.8 ± 0.6
	Feces	3.82	1.15	0.80	6.3		195.2	13.24	11.9
	ISM	1.79 ± 166.4	0.25 ± 135.5	0.08 ± 0.01	7.0 ± 0.2	9.6 ± 0.56		16.4 ± 0.3	14.3 ± 0.3
4	Inoculum	0.244 ± 0.01	0.01 ± 0.01	0.14 ± 0.02	7.6 ± 0.0			14.5 ± 0.3	12.4 ± 0.3
	Feces	3.99	1.05	0.65	6.64		173.5	13.22	11.8
	ISM	1.16 ± 0.08	0.24 ± 0.03	0.12 ± 0.11	7.2 ± 0.1			17.0 ± 0.6	14.8 ± 0.5
5	Inoculum	NA	NA	NA	7.3 ± 0.3		NA	15.2 ± 0.3	13.4 ± 0.5
	Feces	4.31	1.10	0.78	5.1		173.5	12.8	11.3
	ISM	0.86 ± 0.07	0.23 ± 0.03	0.19 ± 0.04	7.0 ± 0.1			18.7 ± 0.7	16.5 ± 0.7
6	Inoculum	0.13 ± 0.01	10.02 ± 0.0	0.0 ± 0.0	7.5 ± 0.5			16.8 ± 0.1	14.6 ± 0.1
	Feces	3.48	1.47	2.37	6.5		183.2	12.8	11.3
	ISM	0.56 ± 0.13	0.11 ± 0.05	0.11 ± 0.03	7.0 ± 0.2			19.3 ± 0.5	17.0 ± 0.5
7	Inoculum	0.18 ± 0.04	0.04 ± 0.0	0.06 ± 0.01	7.5 ± 0.1			17.6 ± 0.3	15.3 ± 0.2
	Feces	3.69	1.00	0.72			183.2	12.8	11.3
	ISM	0.35 ± 0.13	0.05 ± 0.01	0.06 ± 0.01	7.0 ± 0.2	9.4 ± 0.42		20.7 ± 0.4	18.2 ± 0.4
8	Inoculum	0.18 ± 0.03	0.03 ± 0.00	0.21 ± 0.03	7.6 ± 0.1			18.7 ± 0.3	16.2 ± 0.3
	Feces	1.54	0.46	0.26	6.95	5.5	184.2	12.53	11.2
	ISM	0.93 ± 0.06	0.28 ± 0.03	0.29 ± 0.03	7.1 ± 0.1			20.7 ± 1.3	18.0 ± 0.9
9	Inoculum	0.13 ± 0.02	0.03 ± 0.01	0.18 ± 0.02	7.6 ± 0.1			19.5 ± 0.1	17.0 ± 0.1
	Feces	1.54	0.46	0.26	6.95	5.5	184.2	12.53	11.2
	ISM	0.59 ± 0.08	0.11 ± 0.04	0.23 ± 0.04	7.1 ± 0.1			21.5 ± 0.4	19.0 ± 0.4
10	Inoculum	0.18 ± 0.04	0.05 ± 0.02	0.11 ± 0.03	7.7 ± 0.1			20.0 ± 0.2	17.4 ± 0.2
	Feces	5.0	1.34	2.22	6.17		220.5	16.18	14.3
	ISM	0.53 ± 0.11	0.05 ± 0.01	0.17 ± 0.03	7.2 ± 0.1			21.4 ± 0.4	18.7 ± 0.3
11	Inoculum	1.15 ± 0.03	0.03 ± 0.02	9.9 ± 17.1	7.7 ± 0.1	9.7 ± 0.32		20.4 ± 0.4	17.6 ± 0.4
	Feces	5.0	1.34	2.22	6.17		220.5	16.18	14.3
	ISM	0.23 ± 0.04	0.03 ± 0.02	0.0 ± 0.0	7.1 ± 0.3	9.6 ± 0.32		22.0 ± 0.7	19.3 ± 0.7
1-11	Wheat straw	−	−	−	−	−	1097	89.0 ± 0.0	85 ± 0.1

*ISM* inoculum-substrate mixture

### Bioreactor

**Fig. 1:**
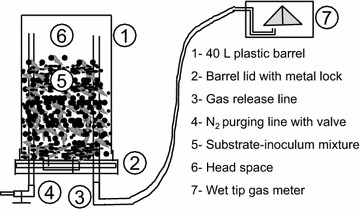
Schematic diagram of the dry anaerobic digester

A triplicate of 40-L cylindrical (0.312 m in diameter × 0.520 m in height) plastic barrels bioreactors were set-up and operated as a SBR at a TCL of 21 days in a temperature controlled room (20 °C). The reactors (Fig. 1) were fitted with two gas lines; one to purge the headspace with nitrogen gas immediately after feeding/loading the substrate to expel O_2_ and initiate an anaerobic environment inside reactors; and the second to release and quantify the biogas produced. The barrel was kept upside down after it has been filled so that the lixiviate works as a water seal around the barrel’s lid to ensure gas tightness.

### Inoculum and substrate

The initial inoculum was obtained from a laboratory scale (40 L) psychrophilic (20 °C) anaerobic sequence batch reactor fed with fresh dairy manure and wheat straw (27 % TS). Fresh feces from dairy cows was collected at the experimental farm of the DSRDC. Feces was collected on wood boards, to avoid dilution with urine, transferred into a plastic drum, stored at 4 °C, before being fed to the reactors. Wheat straw was harvested at the DSRDC`s experimental farm during fall 2011 and fall 2012 and chopped (3 mm) using a laboratory mill (Thomas Wiley Laboratory Mill Model 4, Arthur H. Thomas Company, Philadelphia, PA, USA). Wheat straw and cow feces were mixed manually to obtain the desired substrate TS content (35 %) while maintaining the design OLR of 3.0 g TCOD kg^−1^ inoculum day^−1^.

### Organic loading rate

The OLR has been calculated based on the masses of VS and TCOD of the substrate fed (Table 2). OLR was expressed in g of TCOD fed per kg of inoculum per day, and g of total VS fed per kg inoculum per day. The OLR expression based on mass of inoculum is more suitable for solid substrate in dry anaerobic digestion since measuring the volume is difficult because it is a function of the density or the degree of compaction and water content. The inoculum to substrate ratio (ISR; based on mass of VS) ranged between 4.06 and 4.47. The percentage of substrate-to-inoculum (wet mass-based) ranged between 13.3 and 17.8 %.

### Sampling

The inoculum-substrate mixture (ISM) was sampled on day (0) immediately after feeding. Samples were also taken on day 7 by opening the reactor, mixing its content manually for 5 min to ensure that the sample is representative of the reactor content. The reactor content was also sampled at the end of the treatment cycle (on day 21); this sample represented the digestate (effluent) of which 6 kg were used as inoculum in the next cycle. The gas samples were taken with a 10 mL plastic syringe through a gas sampling port sealed with septa and installed on the gas line mid-way between the barrel and the wet tip gas meter.

### Biogas measurement

Biogas volume produced was measured daily using calibrated wet tip gas meters while the biogas components (CH_4_, CO_2_ and H_2_S) were determined weekly using a Hach Carle 400 AGC gas chromatograph (Model 04131-C, Chandler Engineering, Houston, TX, USA) configured for the application 131-C. The GC setup, protocol and calibration, detection levels, and relevant details were reported elsewhere. Methane (CH_4_) production is reported in normalized litres (NL CH_4_), i.e., the CH_4_ volume produced was corrected to standard temperature and pressure (STP) (273°K; 1 atm.).

### Analytical methods

Samples were collected from each bioreactor and analyzed weekly for volatile fatty acids (VFAs), TS, VS, and pH. The TCOD was determined before and after each treatment cycle. TCOD, TS, VS, alkalinity and pH were determined using standard methods (APHA 1992). Alkalinity: method number 2320B used potentiometric titration to preselected pH 4.38, total solids: method number 2540B, Volatile solids method number: 2540E. VFA: method number 5560D.

The concentrations of individual VFAs including acetic, propionic, butyric, isobutyric, butyric, valeric and isovaleric acids have been measured using Perkin Elmer gas chromatograph (GC) model 8310 (Perkin Elmer, Waltham, Mass.) equipped with an autosampler to facilitate the analysis, fitted with FID, and equipped with a J&W Scientific DB-FFAP high resolution column (30 m × 0.53 mm × 1.00 μm; Chromatographic Specialties Inc., ON, Canada) (Massé et al. 2003). Helium, flowing at 9.5 mL min^−1^, was employed as the carrier gas. The injector temperature was maintained at 200 °C, while the detector temperature was set at 250 °C.

### Fiber analysis

The complex substrate (cow feces and wheat straw) were subjected to fiber analysis to determine their content of cellulose, hemicellulose, and lignin. Hemicellulose can be calculated as the difference between neutral detergent fiber (NDF) and acid detergent fiber (ADF), cellulose as the difference between acid detergent fiber and acid detergent lignin (ADL) (Bauer et al. 2009; Saady and Massé 2013).

## Results

The percent of H_2_S in the biogas was less than 0.06 % in all samples of gas analyzed during the successive cycles. Different cow feces batches sourced from the same dairy barn at DSRDC, Sherbrooke, QC, Canada were used during the 11 cycles as indicated in Table 2. The overall average of the fiber components content (dry matter-based) for all cycles was 24.6 ± 1.4 % (cellulose), 21.2 ± 3.7 % (hemicellulose), and 12.2 ± 1.6 % (lignin). Wheat straw fibers were composed of cellulose (38.61 %), hemicellulose (25.14 %) and lignin (7.3 %); the same batch of wheat straw has been used during all cycles.

### Methane production

**Fig. 2:**
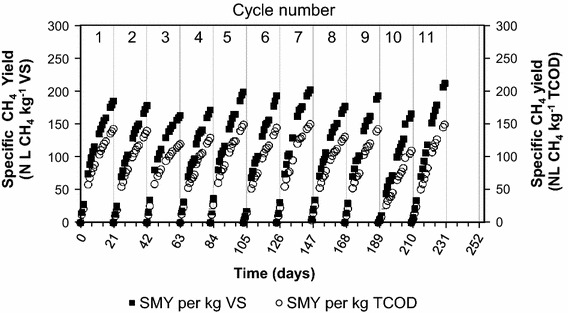
Specific methane yield profiles for psychrophilic dry anaerobic digestion of cow feces and wheat straw (35 % TS)

The performance of psychrophilic anaerobic bioreactors has been evaluated in 11 successive cycles. Methane production profiles were expressed as average specific SMY of the triplicate bioreactors are shown in Fig. 2. The SMYs calculated during the successive cycles are given in Table 3. The SMY has been given per unit mass of VS and TCOD. The profiles of SMY (Fig. 2) showed stable and reproducible methane production with no lag-phases, inhibition, or discontinuation.

**Table 3:** Rate and specific methane yield for the psychrophilic dry anaerobic digestion of cow feces and wheat straw (35 % TS)

Cycle	Cow feces TCOD (g kg^−1^)	Cow feces TCOD/VS	Feed TCOD/VS ratio	SMY (NL CH_4_ kg^−1^ VS)	SMY (NL CH_4_ kg^−1^ TCOD)	Rate of CH_4_ production (NL CH_4_ kg^−1^ VS day^−1^)
1	147.6	1.3	1.3	184.6 ± 14.4	142.3 ± 11.1	8.8 ± 0.7
2	148.0	1.2	1.3	177.7 ± 24.4	139.0 ± 19.1	8.5 ± 1.2
3	195.2	1.6	1.4	213.9 ± 13.6	155.9 ± 9.9	10.2 ± 0.6
4	173.9	1.5	1.3	173.4 ± 17.8	128.6 ± 9.9	8.3 ± 0.9
5	173.9	1.5	1.3	198.2 ± 20.6	148.9 ± 10.9	9.4 ± 1.0
6	173.9	1.5	1.3	200.9 ± 9.9	144.2 ± 12.0	9.6 ± 0.5
7	184.2	1.6	1.4	186.3 ± 28.6	149.6 ± 25.8	8.9 ± 1.4
8	184.2	1.6	1.4	165.7 ± 5.4	125.1 ± 4.9	7.9 ± 0.3
9	184.2	1.6	1.4	191.3 ± 5.6	142.2 ± 3.9	9.1 ± 0.3
10	220.5	1.5	1.4	165.4 ± 9.8	109.8 ± 13.0	7.9 ± 0.5
11	220.5	1.5	1.4	209.1 ± 21.4	149.0 ± 10.9	10.0 ± 1.0

The treatment cycle length in all cycles was 21 days

The numbers given are averages and standard deviations of triplicates bioreactors

The OLR was maintained at 3.0 g TCOD kg^−1^ inoculum day^−1^ equivalent to (2.2 ± 0.1 g VS kg^−1^ inoculum day^−1^)

The SMY in Table 3 is based on the quantity of VS fed (cow feces and wheat straw)

Based on the total VS fed (cow feces plus wheat straw), the average SMY calculated for the triplicate bioreactors ranged between 165.7 ± 5.4 and 213.9 ± 13.6 NL CH_4_ kg^−1^ VS during the 11 successive cycles operated at OLR of 3.0 g TCOD kg^−1^ inoculum day^−1^ (equivalent to 2.2 ± 0.1 g VS kg^−1^ inoculum day^−1^) and TCL of 21 days (Fig. 2; Table 4). The overall average SMY of the 11 successive cycles was 187.9 ± 16.5 NL CH_4_ kg^−1^ VS (139.9 ± 13.1 NL CH_4_ kg^−1^ TCOD).

**Table 4:** Comparative performance of dry anaerobic digestion of cow manure and wheat straw

Substrate and inoculum	Temperature (°C)	TS (%)	ISR	OLR (g TCOD kg^−1^ inoculum day^−1^)	Retention time (days)	SMY (NL CH_4_ kg^−1^ VS)	References
Cow feces and wheat straw	20	35	4.1–4.5	3.0	21	187.9 ± 16.5	This study
Cow feces and wheat straw	20	27	3.0	3.0	21	182.9 ± 16.9	Massé and Saady (2015c)
Cow feces and wheat straw	20	27	2.5	4.0	21	187.3 ± 18.4	Saady and Massé (2015)
Cow feces and wheat straw	20	27	1.7	5.0	21	163.6 ± 39.5	Saady and Massé (2015)
Cow feces and wheat straw	20	27	1.4	6.0	21	150.8 ± 32.9	Saady and Massé (2015)
Cow feces and wheat straw	20	27	1.7	7.0	21	147.1 ± 17.2	Massé and Saady (2015a)
Cow feces and wheat straw	20	27	1.4	8.0	21	143.2 ± 11.7	Massé and Saady (2015a)
CM:WWS (2:3 mass ratio)	35	16	0.2	0.35^a^	63	328	Li et al. (2011a)
Aerobically pre-treated SM, agricultural residues	35	28	NR	0.28^b^	130	55	Di Maria et al. (2012)
	35	28	NR	0.28^b^	65	22	
DM and SG	55	15	0.2	NR	62	28^c^	Ahn et al. (2010)
Rice straw and corn stover inoculated with (1:1) sewage sludge: pig manure		15		NR	156	346	Sun et al. (1987)
		20		NR	156	339	
	26–28	25	0.2	NR	168	382	
		30		NR	198	423	
		35		NR	198	34	
85 % BM plus 15 % GS	35	28	NR	0.9	100	227^c^	Schäfer et al. (2006)
Beef manure plus straw	32	18	NR	3.2	28	181^c^	Schäfer et al. (2006)
SM, turnip rape straw and wheat straw	35	16	NR	NR	120	122^c^	Schäfer et al. (2006)
DM, straw, and oat husk	38	17	NR	3.4	22	160^c^	Schäfer et al. (2006)
DM, straw, and oat husk	38	17	NR	4.1	22	84	Schäfer et al. (2006)
Fresh HM and straw	37	20	0.2	NR	28	146	Kusch et al. (2008)
	37	20	0.2	NR	42	175	
	37	20	0.2	NR	72	208	

*BM* beef manure, *CM* cow manure, *DM* dairy manure, *GS* grass silage, *HM* Horse manure, *PM* poultry manure, *SG* switchgrass, *SM* swine manure, *WWS* wastewater sludge, *NR* not reported

^a^OLR units are kg TCOD kg ^−1^ VS

^b^OLR units are kg VS substrate kg ^−1^ VS inoculum

^c^SMY units are L CH_4_ kg^−1^ VS

The average specific CH_4_ production rate (NL CH_4_ kg^−1^ VS day^−1^) of the replicate bioreactors ranged between 7.9 ± 0.3 (cycle 8) and 10.2 ± 0.6 (cycle 3). The overall average of the specific CH_4_ production rate for the 11 successive cycles was 9.0 ± 0.8 NL CH_4_ kg^−1^ VS day^−1^.

### Volatile fatty acids (VFAs) production

**Fig. 3:**
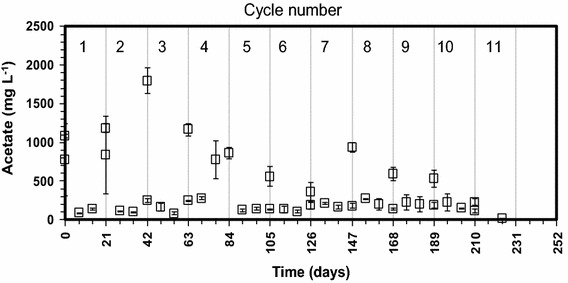
Acetic acid profile for the cow feces and wheat straw psychrophilic dry anaerobic digestion at 35 % total solids in feed

Profiles of acetic, propionic, and butyric acids produced during the successive cycles of PDAD were similar but at different concentration levels. The acetate profile is shown in Fig. 3. Throughout the successive cycles, acetic acid concentration peaked immediately after feeding to levels between 1000 and 1500 mg L^−1^ but was consumed within a week in all replicate bioreactors and its concentrations were maintained within 150 ± 50 mg L^−1^ indicating that methanogenesis reaction from acetate was not a rate limiting step after the first week. Similarly, propionic acid peaked to levels between 500 and 600 mg L^−1^ after feedings and was consumed within a week to levels close to the detection limits of the instrument (25 ± 10 mg L^−1^).

The profile of propionic acid in the replicate bioreactors was similar to that of acetic acid. Butyric acid peaked also to levels between 300 and 500 mg L^−1^ after feedings and was consumed within a week to levels close to the detection limits of the instrument (25 ± 10 mg L^−1^). The concentrations of other volatile fatty acids (isobutyric-, iso-valeric-, and valeric-acid) were less than 50 mg L^−1^ immediately after feeding and less than 25 mg L^−1^ during the remaining time of the successive cycles.

### Solids profiles and removal

The profiles of the TS and VS in the replicate bioreactors during the successive cycles were identical (data not shown). Starting from cycle 7 the variation in the inoculum TS between successive cycles stabilized below 5 % (during cycle 10 and 11 it was 2.6 and 2.0 %, respectively). Similarly, starting from cycle 7 the variation in the inoculum VS between successive cycles stabilized below 5 % (during cycle 10 and 11 it was 2.4 and 1.1 %, respectively). During the last three cycles (9, 10, and 11) the inoculum TS stabilized around 20.0 ± 0.5 % while the inoculum VS stabilized around 17.3 ± 0.3 %. The reduction in the feed-to-effluent TS ranged between 19.6 and 42.1 % while the reduction of VS ranged from 17.2 to 38.3 %. The overall average reduction in the TS and VS were 31.7 ± 6.7 and 29.5 ± 7.6 %, respectively.

## Discussion

Due to the nature of substrate fed which contained lignocellulose components and the relatively short treatment cycle length (21 days) the cumulative SMY did not reach plateau. Therefore, residual substrate has been carried over from cycle to cycle.

Although the OLR (3.0 g TCOD kg^−1^ inoculum day^−1^), the feed total solids (35 %), and the TCL (21 days) have been maintained the same during the cycles 1–11, respectively, the SMY fluctuated from cycle to cycle likely due to the variable quality of the cow feces fed; the quality of the biodegradable organic materials in cow feces is a function of cow diet composition, as well as the quality of the inoculum. Notice that the yield achieved during the first cycle (184.6 ± 14.4 NL CH_4_ kg^−1^ VS) was similar to the overall average SMY for the 11 successive cycles (187.9 ± 16.5 NL CH_4_ kg^−1^ VS) which indicates a relatively fast start up likely because the inoculum was well-adapted to the psychrophilic conditions and the high solids content (35 %) for long time.

Generally, the consistency of the performance of the replicate bioreactors indicates a rapid start-up, stable reproducible process during the 231 days of operation which could be attributed to the increase in the population of the microorganisms due to the increase in the fiber in the biomass.

The dry matter of wheat straw is composed of cellulose (38.61 %), hemicellulose (25.14 %) and lignin (7.3 %). Complete degradation of wheat straw during anaerobic digestion requires long retention time because its hydrolysis is a rate limiting step since it is in solid or particulate form (Myint and Nirmalakhandan 2006). However, the solid retention time (SRT) has been decoupled from the TCL by operating the bioreactors as sequential batch reactors with SRT of about 169 days. Notice that wheat straw formed 74.5 ± 3.8 % of the VS in feed, 78.2 ± 3.5 % of the feed fibers, 73.1 ± 4.0 % of the TS in feed, 71.3 ± 4.5 % of the TCOD fed, and 29.3 ± 2.2 % of the mass of feed (data not shown).

The authors of this study have recently published on psychrophilic dry anaerobic digestion of cow feces and wheat straw (27 % TS in feed) at OLR 3.0–8.0 g TCOD kg^−1^ inoculum day^−1^ (Massé and Saady 2015a, b, c; Saady and Massé 2015). Nevertheless, data on performance of PDAD of cow feces and wheat straw is not available in the accessible literature; therefore, the results have been compared to the performance of mesophilic and thermophilic DAD of various substrates as well as to the data recently published by the authors on PDAD of cow feces and wheat straw at feed TS of 27 % (Table 4). Interestingly, the average SMY (187.9 ± 16.5 NL CH_4_ kg^−1^ VS) obtained from feed TS of 35 % applied at OLR of 3.0 g TCOD kg^−1^ inoculum day^−1^ was statistically not different from the SMYs (182.9 ± 16.9 and 187.3 ± 18.4 NL CH_4_ kg^−1^ VS) obtained from feed TS 27 % and OLR of 3.0 and 4.0 g TCOD kg^−1^ inoculum day^−1^, respectively. The average yield of 187.9 ± 16.5 NL CH_4_ kg^−1^ VS of cow feces and wheat straw (35 % TS at OLR 3.0 g TCOD kg^−1^ inoculum or 2.28 ± 0.14 kg VS fed kg^−1^ inoculum day^−1^) obtained in this study after 21 days of psychrophilic (20 °C) incubation during the 11 successive cycles is greater than the yield 160 NL CH_4_ kg^−1^ VS of dairy manure, straw, and oat husk (TS 17 % at OLR of 3.4 g VS kg^−1^ day^−1^) reported by Schäfer et al. (2006) for Jarna biogas plant in Sweden which operates at 38 °C and retention time of 22 days. Notice that the data reported from Jarna plant is for a steady-state condition where the inoculum was adapted to the substrate and the operation condition for 3 years at the time of the study reported by Schäfer et al. (2006). The SMYs from cow feces and wheat straw at a TCL of 21 days in any of the PDAD eleven successive cycles (TS 35 %) obtained in this study were higher than 28 L CH_4_ kg^−1^ VS of dairy manure and switch grass (15 % TS) obtained by Ahn et al. (2010) during 62 days of thermophilic (55 °C) incubation. The difference between Ahn et al. (2010) and this study results demonstrate the importance of prolonged adaptation period and the importance of ISR. Ahn et al. (2010) used low ISR (0.2) and non-adapted inoculum in short time study while in this study the inoculum was adapted during around 36 months to stepwise increase in the feed’s total solids (data not shown) and sufficient quantity of inoculum has been used (VS-based ISR ranged between 1.7 and 3.8). The average SMY (188 ± 17 NL CH_4_ kg^−1^ VS) is similar to 181 L CH_4_ kg^−1^ VS of beef manure and straw (TS 18 % and OLR of 3.2 g VS kg^−1^ day^−1^) at 32 °C and retention time of 28 days reported previously (Schäfer et al. 2006) (Table 4). Compared to Schäfer et al. (2006) result, the current study demonstrated an increase of 94 % in the feed total solids and reduction in the treatment cycle length by 25 % while saving the energy consumed in heating the bioreactor (to increase the temperature from 20 to 37 °C); these improvements translate into 35 and 25 % reduction in the required volume of the bioreactor, respectively, while at the same time cutting the reactor heating expenses by operating at psychrophilic condition. Notice that the high yields (>250 NL CH_4_ kg^−1^ VS fed) reported by Li et al. (2011a) was for mesophilic anaerobic digestion of cow manure and wastewater sludge (16 % TS) in 63 days of TCL. Similarly, the yield (339–423 NL CH_4_ kg^−1^ total VS) reported by Sun et al. (1987) in Table 4 have been obtained for long retention times (156–198 days) and low OLR (0.35 kg TCOD kg^−1^ inoculum day^−1^) in mesophilic anaerobic digestion of rice straw and corn stover (TS 15–30 %). Interestingly, these researchers reported a very low SMY (34 NL CH_4_ kg^−1^ VS fed) for the same substrate and experimental conditions at TS 35 %. Achieving a stable dry anaerobic digestion of cow manure and wheat straw at psychrophilic condition and feed TS of 35 % and OLR of OLR 3.0 g TCOD kg^−1^ inoculum or 2.28 ± 0.14 kg VS fed kg^−1^ inoculum day^−1^ over long-term start-up operation is a significant improvement given that 30 % TS has been recently identified as a threshold above which methanogenesis was strongly inhibited for cardboard batch anaerobic digestion at 35 °C (Abbassi-Guendouz et al. 2012).

### Inoculum-to-substrate ratio

The ISR used in this study might be relatively high compared the ISR of 0.2 which has been used by some studies (Ahn et al. 2010; Kusch et al. 2008; Sun et al. 1987; Li et al. 2011a) (Table 4). However, the high total solids content of the substrate (35 %), psychrophilic (20 °C) condition which decreases the rate of biological activity of microorganisms, and the recalcitrant nature of lignocellulose components in wheat straw and cow feces all justify the relatively high ISR used in this work. It is worthwhile to notice that comparing 63, 62, and 168 days of cycle length to 21 days shows the novelty of the present study. Moreover, the SMY (28 L CH_4_ kg^−1^ VS) for 15 % TS dairy manure and Switchgrass digestion during 62 days of incubation at 55 °C was a sheer failure (Ahn et al. 2010); similarly 156–198 days (5.2–6.6 months of incubation) (Sun et al. 1987) is far from being practical and economic. The only promising result from the other studies cited in Table 4 would be that obtained SMY of 146 NL CH_4_ kg^−1^ total VS in 28 days at 37 °C from 20 % TS fresh horse manure and straw (Kusch et al. 2008). Noting that this study increased the feed TS by 75 % and decreased the temperature from 37 to 20 °C justifies the relatively high ISR used.

Although the OLR used in this study (2.2 ± 0.1 g VS kg^−1^ inoculum day^−1^) is comparable that used in farm anaerobic digestion plants in Europe (3.0 g TVS L^−1^ reactor day^−1^) the TCL in this study is less by 50 % (Bolzonella et al. 2011). The specific methane yields obtained in this study provide evidence that the start-up of PDAD of cow manure and straw is practically feasible at TS 35 % within 147–210 days and is as efficient as mesophilic DAD given that a sufficient quantity of well-acclimatized inoculum is used.

**Fig. 4:**
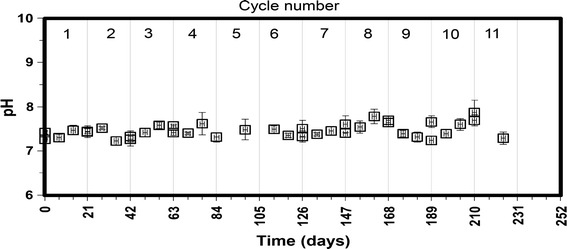
pH profile for the cow feces and wheat straw psychrophilic dry anaerobic digestion at 35 % total solids

The profiles of the VFAs concentration and the high level of methane production during the successive cycles indicate that acetogenic and methanogenic reactions proceeded fairly well. The relative stability of the pH profile around 7.2 ± 0.6 (Fig. 4) was due the sufficient alkalinity which ranged between 9.6 ± 0.56 and 9.4 ± 0.42 g CaCO_3_ L^−1^ for the inoculum-substrate mixture immediately after feeding. The alkalinity of the inoculum at the end of cycles ranged between 9.7 ± 0.32 and ±11.8 ± 0.65 g CaCO_3_ L^−1^ (Table 2).

## Conclusions

A successful start-up of psychrophilic (20 °C) dry anaerobic digestion of cow feces with wheat straw at 35 % total solids in feed has been demonstrated within 147–210 days (7–10 successive cycles of 21 TCL each). An average specific methane yield (SMY) of 188 ± 17 NL CH_4_ kg^−1^ VS fed (140 ± 13 NL CH_4_ kg^−1^ TCOD fed) has been achieved in 21 days treatment cycle length (TCL) at feed TS of 35 % and OLR of 3.0 g TCOD kg^−1^ inoculum day^−1^ in a laboratory scale sequencing batch reactor inoculated with psychrophilic anaerobic mixed culture. A maximum SMY of 214 ± 14 NL CH_4_ kg^−1^ VS fed (156 ± 10 NL CH_4_ kg^−1^ COD fed) with a maximum CH_4_ production rate of 10.2 ± 0.6 NL CH_4_ kg^−1^ VS day^−1^ have been accomplished depending on the quality of cow feces fed. The process was stable with an overall average 30.9 ± 6.6 % reduction in the volatile solids. The measured volatile fatty acids concentrations indicated that hydrolysis was the reaction limiting step. The results indicate that dry anaerobic digestion of dairy cow manure and wheat straw at feed total solids of 35 % and TCL of 21 in sequential batch reactor is feasible and as efficient as mesophilic operation.
